# Emergency department outcome of elderly patients assisted by professional home services, the EPIGER study

**DOI:** 10.1186/s12877-020-01742-1

**Published:** 2020-09-21

**Authors:** Anne-Laure Feral-Pierssens, Gustave Toury, Fatima Sehimi, Nicolas Peschanski, Saïd Laribi, Amélie Carpentier, Magali Kraif, Clément Carbonnier, François-Xavier Duchateau, Yonathan Freund, Philippe Juvin, the EPIGER IRU-SFMU study group, Patrice Serre, Aurélie Arnaud, Edouard Dugat, Céline Occelli, Thomas Lanoux, Edouard Jacquet, Céline Moretto, Arnaud Devillard, Aurélia Bordais, Olivier Maurin, Magali Kraif, Aurélie Caffier, Jonathan Duchenne, Stéphane Gillet, Ester Milojevitch, Cindy Tissier, Sunde Kilic, Véronique Uchlinger, Nicolas Gonzalez, Vincent Lacoste, Sébastien Pouzoulet, Abdo Khoury, Philippe Marguet, Claude Zamour, Arnaud Proust, Anastasia Dessena, Frédéric Saura, Aurélie Schindler, Emilie Gelin, Ludivine Tendron, Isabelle Cailleux, Marie-Hélène Basso, Vanessa Houze-Cerfon, Julie Oudet, Bernard Ah-Koon, Thierry Babet, Justine Bereau, Rishad Vally, Emilie Souchaud, Catherine Scouarnec, Olivier De Stabenrath, Olivier Vuillot, Isabelle Giraud, Laurent Bebien, Nicolas Chauvel, Thomas Le Normand, Cécile Rouchy, Isabelle Arnault, Vivien Brenkmann, Damien Viglino, Ernest Maiello, Olivier Matas, Rémy Lemarchand, Yves Duffait, Cécile Bonhomme, Mikaël Martinez, Alain Viallon, Quentin Legoff, Benjamin Blonstein, Pierre-Arnaud Fort, Oriane Vicenzi, Valérie Ruche, Anthony Millet, Tahar Chouihed, Daniel Baugnon, Nathalie Daniel, Bertrand Boulanger, Julien Galant, Henri Le Hot, Christophe Rothmann, Isabelle Guenot, Mathieu Cochonneau, Nadia Smaiti, Patricia Lachery, Eric Wiel, Sylvain Thiriez, Lila Abdelli, Amélie Carpentier, Rachid Kasdali, Thierry Ramaherison, Thomas Guidez, Charles Bailly, Fabien Poher, Annick Idrissi, Karine Humbert, Paul Andregnette, Daniel Pic, Nicolas Dublanchet, Géraldine Giroud, Guillaume N’Guyen, Laure Jainsky, Marc Lacrouts, Marie-Pierre Liepa, Gaëlle Esturoune, Arnaud Ximenes, Ialasoa Randrianasolo, Mélanie Mathe, Hélène Chable, Géraldine Le Cardinal, Anne-Marie Zix-Minni, Pierrick Le Borgne, Fanny Schweitzer, Kasarra Ben Hammouda, Jacques Schmitt, Gaëlle Compte, Marine Delaroche, Christian Di Filippo, Véronique Potinet, Olivier Regal, Alireza Nahani, Jacques Faivre, Teddy Sturiale, Mohammed Touil, Mario Di Rollo, Olivier Laine, Mathieu Gerain, Marc Latappy, François-Xavier Ageron, Claire Vallenet, Agathe Leleu, Morgan Blandin, Anne-Laure Paquet, Marie-Laurence Fievet-Brochot, Erwin Hansconrad, Benoît Vivien, Alessandra Principe, Pierre-Clément Thiebaud, Eloïse Trabattoni, Eric Burggraff, Emmanuel Boust, Valérie Massol, Xavier Benet, Quentin Foubert, Benoît Jardel, Mélanie Roussel, Luc-Marie Joly, Mariane Ovtcharenko, Karim Bedrici, Mohamed Abdeljaouad, Carole Mauger-Briche, Laurence Berton, Ludovic Dalle, Mathieu Violeau, Loïc Amizet, Fanny Fontaine, Anaïs Colonna, Jean Tida, Emelyne Cwicklinski, Philippe Fradin, Christine Vallejo, Lotfi Frigui, Samia Bregigeon, Muriel Porche, Arnaud Le Jan, Jean-Philippe Desclefs, Hery Andrianjafy, Laura Wajzer, Ta Trung Hung, Sébastien Beaune, Hugo Lenglet, Gaëlle Le Bail, Anna Bouchara, Marie-Clément Kouka, Mathias Wargon, Steven David, Mohamed Khalid, Catherine Phlippoteau, Stéphane Diez, Jean Sende, Xavier Baermann, Catherine Legall, Aurélie Fehre, Célia Etiennar, Nathalie Roudiak, Julie Talfournier, Chloé Lefebvre, Yann-Erick Claessens, Pierre-Nicolas Carron, Fabrice Dami, Esther Popotte, Ahmed Belkouch, Jean-Marc Pujo

**Affiliations:** 1 Emergency department, Georges Pompidou european hospital, Assistance Publique Hôpitaux de Paris, Paris, France; 2 IMProving Emergency Care academic federation, Paris, France; 3 CR-CSIS, Université de Sherbrooke, Longueuil, Québec, Canada; 4 Emergency department, Saint-Antoine hospital, Assistance Publique Hôpitaux de Paris, Paris, France; 5 Emergency department, Charles Nicolle University Hospital, Rouen, France; 6 Tours University, Tours, France; 7 Emergency department, Tours University Hospital, Tours, France; 8 Emergency department and Emergency Medical Service, Jean-Bernard Hospital, Centre Hospitalier de Valenciennes, Valenciennes, France; 9 Emergency department, La Timone hospital, Assistance Publique des Hôpitaux de Marseille, Marseille, France; 10 Sciences Po, LIEPP, Paris, France; 11 Chaire en fiscalité et finances publiques, Université de Sherbrooke, Longueuil, Canada; 12 Emergency Medical Service, Raymond Poincaré Hospital, Assistance Publique Hôpitaux de Paris, Garches, France; 13 Sorbonne Université, Paris, France; 14 Emergency department, Pitié-Salpétrière hospital, Assistance Publique Hôpitaux de Paris, Paris, France; 15 Université de Paris, Paris, France; 16 Initiative de Recherche aux Urgences, French Society of Emergency Medecine, Paris, France

**Keywords:** Elderly, Healthcare access, Loss of autonomy, Home services, Emergency care

## Abstract

**Background:**

For the elderly population living at home, the implementation of professional services tends to mitigate the effect of loss of autonomy and increases their quality of life. While helping in avoiding social isolation, home services could also be associated to different healthcare pathways. For elderly patients, Emergency Departments (EDs) are the main entrance to hospital where previous loss of autonomy is associated to worst hospital outcomes. Part of elderly patients visiting EDs are still admitted to hospital for having difficulties coping at home without presenting any acute medical issue. There is a lack of data concerning elderly patients visiting EDs assisted by home services. Our aim was to compare among elderly patients visiting ED those assisted by professional home services to those who do not in terms of emergency resources’ use and patients’ outcome.

**Methods:**

A multicenter, prospective cohort study was performed in 124 French EDs during a 24-h period on March 2016.Consecutive patients living at home aged ≥80 years were included. The primary objective was to assess the risk of mortality for patients assisted by professional home services vs. those who were not. Secondary objectives included admission rate and specific admission rate for “having difficulties coping at home”. The primary endpoint was in-hospital mortality. Cox proportional-hazards regression model was used to test the association between professional home services and the primary endpoint. Multi variables logistic regressions were performed to assess secondary endpoints.

**Results:**

One thousand one hundred sixty-eight patients were included, median age 86(83–89) years old,32% were assisted by professional home services. The overall in-hospital mortality rate was 7%. Assisted patients had more investigations performed. Home services were not associated with increased in-hospital mortality (HR = 1.34;95%CI [0.68–2.67]), nor with the admission rate (OR = 0.92;95%CI [0.65–1.30]). Assisted patients had a lower risk of being admitted for “having difficulties coping at home” (OR = 0.59;95%CI [0.38–0.92]).

**Conclusion:**

Professional home services which assist one-third of elderly patients visiting EDs, were not associated to lower in-hospital mortality or to an increased admission rate. Assisted patients were associated to a lower risk of being admitted for «having difficulties coping at home».Professional home services could result in avoiding some admissions and their corollary complications.

**Trial registration:**

Clinicaltrial.gov - NCT02900391, 09/14/2016, retrospectively registered

## Background

Elderly population experiencing loss of autonomy have limited access to ambulatory care and have an increased use of emergency departments (EDs) when an acute disease occurs [1, 2]. EDs remain the main entry of elderly patients into hospitals [1, 3]. In 2017, 40% of French individuals aged 80 years and over had been admitted at least once to hospital, with a reported longer length-of-stay and higher readmission rate for those with loss of autonomy [1, 4–6]. In the ED, the degree of autonomy of elderly patients is a major determinant of patients’ pathway, needed care and outcomes [7–9]. Part of elderly patients visiting EDs are still admitted to hospital for having difficulties coping at home without presenting any clear acute medical issue. The existence of frailty is associated with an increased risk of healthcare facility use, inpatient hospital admission or institutionalization, further worsening of the autonomy level and a three-time increase of 5 years-mortality rate [1, 3, 10, 11].

Previous reports introduced assessment tools for elderly autonomy, which did not investigate nor detailed homecare services received by elderly patients visiting ED. [12–14] These home services can be delivered by relatives (spouse, family, friends) or professional companies. They range from meal delivery to assistance with dressing, bathing, or toileting. These professional home services have been designed to mitigate the consequences of loss of autonomy for elderly patients still living at home, increase their quality of life and avoid social isolation. Those home services become inevitable when loss of autonomy is increasing with the burden of needed care. However, despite their development for the last 20 years, there is still an inequal access to them [15–17]. Thus, while emergency physicians take care of elderly patients it is usual care to look for information concerning any existing home services, allowing the assessment of patients’ vulnerability to loss of autonomy and its subsequent consequences. However, there is a lack of data concerning the profile of home services received by the elderly population visiting EDs and their ED resource use and outcomes.

The aim of this study is to compare the outcomes of elderly patients visiting EDs between those assisted by professional homecare services and those who do not.

## Methods

### Study design

A multicenter prospective cohort study was conducted in 124 emergency departments during a 24 h period on March 16th 2016. Our sample comprises both urban and rural centers (2500 to 88,000 visits a year). All participating EDs were members of the French Emergency Medicine Research Network, section of the French Society of Emergency Medicine.

### Study population and data collection

**Fig. 1:**
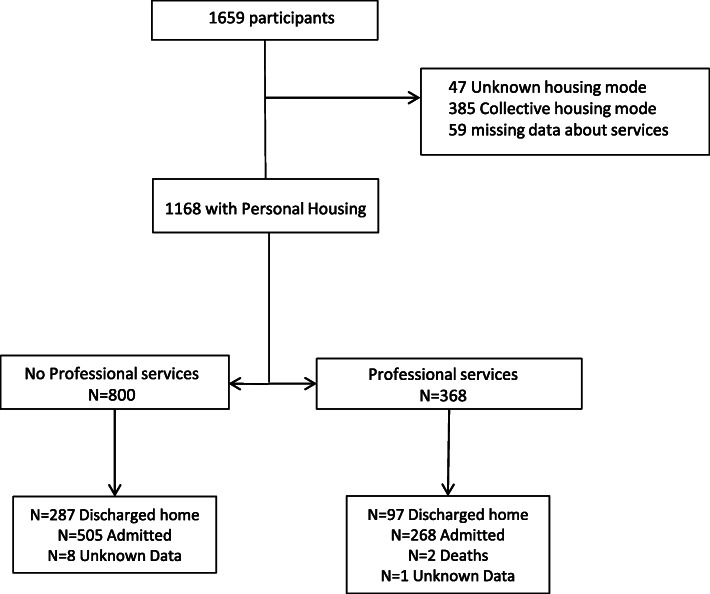
Flow chart of included patients and existing home services

On march 16th 2016, from 9 am to 9 am on march 17th, all consecutive patients aged 80 years and older visiting the ED were included. Patients living in an elderly healthcare facility were not included. Patients with missing data concerning housing mode and home services were excluded (Fig. 1). Data were collected through patients’ and relatives’ interviews, medical examination and computerized medical charts.

### Measurements

#### Autonomy

Two different scales were performed to assess patient’s autonomy: Activities of Daily Living (ADL) Scale and Knaus Classification [13, 18]. Knaus classification is subdivided in four levels: A, prior good health status without functional limitation; B, mild to moderate limitation of activities due to chronic medical illness; C, chronic illness producing serious but not incapacitating restriction of activities; D, severe restrictions of activities due to chronic illness including institutionalized and bedridden patients. ADL score ranges from 0 (full autonomy) to 12 (bedridden patients).

#### Home services

In-home attendance of professional services and their assigned tasks were collected when it concerned cleaning, bathing, dressing, walking, eating or doing groceries. Data concerning technical devices were also collected. Patients were considered as “assisted by home services” when they were assisted by professionals assigned to any task mentioned above except when home services were only dedicated to house cleaning.

#### Emergency department care

The acute illness for which the patient visited the ED was considered as “serious” if one of the following was observed: arriving by medical transportation, Glasgow Coma Score on arrival below 14, organ distress or being moved towards resuscitation bay on arrival. The type of performed investigations tests (laboratory, imaging) and specific administered drugs (analgesic and psychotropic drugs) were collected. Patient’s primary diagnosis was identified by the emergency physician who could specify if admission was due to “difficulties in coping at home”. That last situation referred to an inadequate balance between the patient’s functional resources and the constraints and complications that returning home might have been precipitating without a clear acute medical issue.

Patients were followed-up until hospital discharge or day 30, whichever came first.

### Endpoints

Our primary objective was to assess the risk of mortality for elderly patients assisted by professional home services vs. those who were not. Secondary objectives were to compare ED resource use and ED outcomes between these two categories. The primary endpoint was in-hospital mortality. Secondary endpoints included admission rate after ED visit, admission rate for “having difficulties coping at home” and length-of-stay for admitted patients.

### Statistical analysis

Continuous data were expressed as mean (SD) if normally distributed or median (interquartile range) if not. Categorical data were reported as number and percentage. Comparisons were performed between elderly patients assisted by professional home services and those who did not by using Student t test for continuous data and Chi-square tests for categorical data. All comparisons were 2-tailed, and a *P*-value less than .05 was required to reject the null hypothesis.

Cox proportional-hazards regression model was used to test the association between professional home services and mortality following an ED visit. Adjusting factors were age, comorbidities (history of respiratory disease, polymedication (≥3 daily drugs), Knaus classification, cognitive impairment), serious acute illness and performed investigations. Hazard ratios were calculated with 95% confidence intervals.

Binary multi variables logistic regression models were performed to assess patients’ admission rate after ED visit adjusting for potential a priori confounding factors: age, sex, comorbidities (history of respiratory, cardiovascular or neurological disease, cognitive impairment, psychotropic drugs, Knaus classification, clinical severity and performed investigations). Adjusted odds-ratios and their 95% confidence interval were estimated. Sensitivity analysis were performed adjusting for existing informal caregivers. Statistical analyses were performed with STAT 12 (StatCorpsLP, CollegeStation, Texas USA). We followed STROBE recommendations for reporting observational cohort studies.

### Ethics approval and consent to participate

This study has been approved by our institutional review board and ethics committee (CERHUPO - Comité d’Ethique pour la Recherche des Hôpitaux Universitaires Paris Ouest) (October, 2015), by CCTIRS (Comite Consultatif sur le Traitement de l’Information en matière de Recherche dans le domaine de la Santé) and by CNIL (Commission nationale informatique et liberté) (Record 15.1015bis). According to French law and national guidelines (Law n°94–548 of July 1st 1994 relating to the processing of personal data for the purpose of research in the health field), need for written informed consent was waived.

## Results

Over the study period, 1659 patients aged 80 years and older visited one of 124 participating ED. Among them, 491 patients living in collective housing or with missing data concerning their housing mode were not included (Fig. 1). Overall, 1168 patients were analyzed among whom 368 (32%) where assisted by home services.

### Patients characteristics

Median age of the population was 86 (83–89) years old. Patients assisted by professional home services were older than those who were not: with a median age of 88 (85–91) vs. 85 (82–88) (*P* < 0.001). They also presented with more comorbidities such as history of cardiovascular, respiratory or neurologic diseases. Prevalence of cognitive impairment and polymedication were more frequent in assisted patients. The rate of patients that had registered their personal directives in case of incapacity because of severe illness, was similar between the two groups (Table 1). Among patients assisted by home services, 14% were considered as class A in Knaus classification compared to 48% of patients that were not assisted by any professional services (*P* < 0.001). Similarly, 33% of assisted patients had an ADL score ≥ 6 compared to 9% of patients without home services (*P* < 0.001). In terms of types of caregivers, 55% of patients already assisted by services were also cared by their relatives (Table 1).

**Table 1:** Health characteristics, autonomy assessment and home services of elderly patients visiting EDs according to professional home services’ presence

Characteristics	All patients *N* = 1168 N(%)	No services *N* = 800 N(%)	Services *N* = 368 N(%)	*P*-value^*^
**Age, y.o median (Q1;Q3)**	86 (83;89)	85 (82;88)	88 (85;91)	< 0.001
**Sex ratio**	0.72	0.75	0.65	0.310
**Medical history:**				
None	38(3)	31(4)	7(2)	0.077
Cardio Vascular risk factors	686(59)	467(58)	219(60)	0.714
Cardio Vascular	772(66)	504(63)	268(73)	0.001
Respiratory	198(17)	122(15)	76(21)	0.022
Neurologic	272(23)	152(19)	120(33)	< 0.001
Psychiatric	89(8)	54(7)	35(10)	0.099
Trauma	91(8)	58(7)	33(9)	0.309
Hematology/oncology	212(18)	136(17)	76(21)	0.132
Urology	192(16)	130(16)	62(17)	0.798
Gastro enterology	189(16)	117(15)	72(20)	0.033
≥ 2 categories	496(42)	293(37)	203(55)	< 0.001
**Usual medication**				
None	47(4)	40(5)	7(2)	0.012
≥ 3 daily medications	689(59)	448(56)	241(65)	0.002
Hypertension	744(64)	509(64)	235(64)	0.938
Antiplatelets	424(36)	285(36)	139(38)	0.478
Anticoagulant	273(23)	166(21)	107(29)	0.002
Psychotropics	264(23)	158(20)	106(29)	0.001
**Cognitive impairment**	308(26)	156(20)	152(41)	< 0.001
**Relatives present in the ED**	421(36)	304(38)	117(32)	0.040
**Registered personal directive**	18(2)	12(2)	6(2)	0.775
**Autonomy assessment**				
Living alone	556(48)	346(43)	210(57)	< 0.001
Living as couple	446(38)	340(43)	106(29)	< 0.001
Helping spouse	136(12)	97(12)	39(11)	0.252
**Knaus classification**				
A	437(37)	385(48)	52(14)	< 0.001
B	451(39)	281(35)	170(46)	< 0.001
C	230(20)	97(12)	133(36)	< 0.001
D	4(0.3)	0(0)	4(1)	0.003
**Autonomy assessment:**				
ADL^a^, median (Q1;Q3)	1(0;4)	0(0;1)	3(1;6)	< 0.001
Severe disability (ADL^a^ ≥ 10)	66(6)	29(4)	37(10)	< 0.001
Loss of autonomy (ADL^a^ ≥ 6)	194(17)	73(9)	121(33)	< 0.001
Early disability (ADL^a^ ≤ 4)	877(75)	674(84)	203(55)	< 0.001
Disability for intimate tasks	136(12)	41(5)	95(26)	< 0.001
Walking disability	123(11)	57(7)	66(18)	< 0.001
**Home services**				
**Type of services**				
Professional	368(32)	0(0)	368(100)	
Relatives	621(53)	418(52)	203(55)	0.021
Cleaning only	181(16)	181(23)	0(0)	
Technical help	220(19)	77(10)	143(39)	< 0.001
**Tasks dedicated to caregivers:**				
Cleaning	599(51)	298(37)	301(82)	< 0.001
Groceries	369(32)	128(16)	241(66)	< 0.001
Bathing	342(29)	81(10)	261(71)	< 0.001
Clothing	232(20)	55(7)	177(48)	< 0.001
Walking	79(7)	16(2)	63(17)	< 0.001
Eating	182(16)	50(6)	132(36)	< 0.001
Delivering meals	138(12)	41(5)	97(26)	< 0.001
Telealarm	119(10)	43(5)	76(21)	< 0.001
Walking device	344(29)	159(20)	185(50)	< 0.001
Nurse care	131(11)	54(7)	77(21)	< 0.001

*P-value when comparing patients benefitting from professional home care and those who do not. ^a^ *ADL* Activity of Daily Living. Score ranging from 0 (full autonomy) to 12 (bedridden)

On arrival, elderly patients assisted by home services presented similar severity signs of acute illness than patients without any professional support (16% vs. 14%, *P* = 0.343). However, assisted patients benefitted from more investigations than patients without home services (88% vs. 78% for biology tests (P < 0.001) and 72% vs. 62% for imaging tests (*P* = 0.001). Trauma was the main cause for ED visit for both groups (Supplemental Table 5).

### Main results

The in-hospital mortality rate was 7%, median length of stay was 8 days (IQR 3–14) (Table 2). Among admitted patients, 30 days mortality rate was 10% for patients assisted by home services vs. 5% for the others (absolute difference 5% [4–6%], *P* = 0.014). After adjustments, the presence of home services was not associated with increased in-hospital mortality (Hazard Ratio = 1.34; 95% Confidence Interval [0.68–2.67]). History of respiratory disease, C or D classes and clinical severity on arrival were associated to an increased in-hospital mortality (Table 3).

**Table 2:** Emergency department outcome of elderly patients according to professional home services’ presence

	All population *N* = 1168 N(%)	No services *N* = 800 N(%)	Services *N* = 368 N(%)	*P*-value
**Outcome after ED visit:**				
Death	2(0.2)	0(0)	2(0.5)	0.037
Admitted	773(66)	505(63)	268(73)	0.001
Admitted for “having difficulties coping at home”	128(11)	80(10)	48(13)	0.124
ED short unit	262(22)	175(22)	87(24)	0.496
Other medical unit	460(39)	290(36)	170(46)	0.001
Intensive care Unit	51(4)	40(5)	11(3)	0.119
Runaway	4(0.3)	3(0.4)	1(0.3)	0.780
Discharged against medical advice	7(0.6)	5(0.6)	2(0.5)	0.868
**On Day 30 among admitted patients:**	*N* = 732	*N* = 480	*N* = 252	
Death	52(7)	26(5)	26(10)	0.014
Still hospitalized	213(29)	133(28)	80(32)	0.298
Discharged home	450(61)	314(65)	136(54)	0.002
Entering Elder facility	17(2)	7(2)	10(4)	0.032
**Length-of-stay:**	*N* = 630	*N* = 415	*N* = 215	
**Days, median (Q1;Q3)**	8 (3;14)	8(3;14)	8(3;14)	0.917

**Table 3:** Hazard ratios of mortality risk by Cox proportion-hazard model

Characteristics	Hazard Ratio [IC95%]
Age	1.01[0.93–1.09]
Respiratory history	2.35 [1.17–4.75]
Cognitive impairment	1.18 [0.59–2.35]
≥ 3 daily medications	0.60 [0.31–1.15]
Knaus C or D	3.99 [1.32–12.1]
ADL^a^ ≥ 6	0.68 [0.30–1.50]
Serious acute illness on arrival	4.19 [2.22–7.91]
Imaging	1.17 [0.55–2.49]
Professional home services	1.34 [0.68–2.67]

Tested variables: age, history of respiratory disease, cognitive impairment, Knaus classification, loss of autonomy (ADL), polymedication, clinical severity, performed investigations, professional home services

^a^*ADL* Activities of Daily Living

Overall, admission rate for all elderly patients was 66%. The admission rate was higher for patients assisted by home services (73% vs. 63%, *P* = 0.001). Emergency physicians considered that 13% of the patients assisted by professional services were admitted for “having difficulties coping at home” vs. 10% for those who were not (*P* = 0.124). Transfer to intensive care unit was necessary for 3% of assisted patients whereas it concerned 5% of patients that were not assisted by home services (*P* = 0.119). There was no difference in hospital lengths-of-stay between the two groups (Table 2).

After multi variable logistic regression, the admission risk after ED visit for elderly patients assisted by professional home services was not different from patients that were not assisted by any professional services (Odd Ratio = 0.92; 95%CI [0.65–1.30]). Assisted patients had a lower risk of being admitted for « having difficulties coping at home » when compared to patients that were not assisted by professional services (OR = 0.59; 95% CI[0.38–0.92]) (Table 4 and supplemental Table 6).

**Table 4:** Odds-ratios (OR) of patients’ outcome after ED visit when comparing elderly patients assisted by professional home services to patients without any professional support

	OR [95%CI]	*P*-value
**ED outcome (*n* = 1168)**		
Admitted	0.92 [0.65;1.30]	0.636
Admitted for “having difficulties coping at home”	0.59 [0.38;0.92]	0.020
Admitted to the ED short unit	0.81 [0.57;1.14]	0.225
Admitted to the Intensive Care Unit	0.70 [0.30;1.64]	0.408
**On Day 30 among admitted patients (*n* = 732):**		
Still hospitalized	1.10 [0.75;1.61]	0.628

Adjusting variables: age, sex, history of respiratory, cardiovascular or neurological diseases, cognitive impairment, psychotropic drugs, Knaus classification, clinical severity and performed investigations

## Discussion

In our sample of 1168 elderly patients from 124 EDs in France, a third were assisted by professional home services while over half of them had access to informal caregivers (such as friends or relatives). There was no association between in hospital mortality and professional home services. Assisted patients had a similar risk of being admitted after ED visit but were associated to a lower risk of being admitted for « having difficulties coping at home ».

The rate of elderly patients assisted by home services matches other published research dealing with elderly population living alone at home [19]. However, the present study detailed professional home services for elderly patients visiting EDs, investigating their dedicated tasks also providing a full profile of this specific population while exploring their emergency resources use and outcomes.

The admission rate reported in this study was superior to those mentioned in literature about geriatric populations but definitions of elderly have varied over the past 20 years depending on publications, which is a limit for comparisons [1, 4, 20]. The DREES survey conducted in 2013 in 736 French EDs noted that elderly patients counted for 12% of the emergency flow and 56% among those were admitted following their visit [20]. However, the present study focussed on visiting patients who lived by themselves at home whereas the DREES survey included elderly patients living in healthcare facilities whose admission rate is usually lower [4]. Otherwise, characteristic features in terms of comorbidities, investigations and primary diagnosis were similar to those noted in literature. Elderly patients are polypathological, come in the forts place for traumatological motives and are given more investigations than younger patients [1, 3, 4]. Finally, the mortality rate as observed in EDs was similar to those noted in other French studies [21, 22].

When comparing both groups, assisted patients suffered more comorbidities (notably cardiovascular, respiratory and neurological) and suffered more frequently from loss of autonomy (whether measured with Knaus classification or the ADL score). After adjusting for those factors, it appears that the in-hospital mortality was not tied to the presence of at home services but was associated to the severity of the acute illness when reaching the ED as well as to the patient’s level of autonomy. Results associating autonomy level and short-term mortality after ED visits was consistent with literature on the subject [7, 8]. The degree of autonomy is already a decisive and essential information in emergency settings in order to allow physicians to make urgent decisions such as engaging in resuscitation manoeuvres [9].

Nevertheless, the risk to be admitted for «having difficulties coping at home» was lower for patients with available home services. This suggests a form of substitutability between at home professional services and the resort to hospital care. However, these services are often regulated outside the scope of health policy. Whereas a number of evaluations of the economic impact of this developing area of activity exist, few studies investigate the sanitary and social benefits of increasing at home support [15, 16]. The French PAERPA program was dedicated to the implementation of a coordination process of already existing home services and its assessment was focussed on various health indicators (visit to EDs, avoidable admissions). Preliminary results did not demonstrate any effect on the different factors under study [23]. However, it did not investigate the implementation of home services for elderly individuals that were lacking home support. The present study found that some patients that were not assisted by home services exhibited a high level of dependency. Combined with the lower rate of admission for «having difficulties coping at home», this suggests on the contrary that public investment bearing on the development of at home support could result in a reduction of part of admissions following ED visits. Adequate care at home for dependency through professional services might have an incidence on the pathway of care for elderly patients and could result in avoiding some admissions and their corollary complications (iatrogenic dependence, lengthening of hospital stay, mortality) [2, 3, 24].

### Limitations

Our study has some limits. In spite of the high number of EDs participating our sampling did not aim at being representative of French EDs as a whole. Thus, the results presented here cannot be extrapolated to other contexts. However, the patients’ profile appears consistent with results achieved in other large-scale studies.

In addition, we noticed differences in the descriptive characteristics of the two compared groups: assisted patients are more likely to present comorbidities, are more often polymedicated and have lower level of autonomy. We tend to compensate this distortion of comparability through multivariable analysis adjusting for these factors even if biases may remain despite the chosen set of adjustment variables. However, even though we noticed differences between the characteristics of the two groups, there are still patients that are not assisted by professional home services presenting with low autonomy level, polymedication and comorbidities; conversely, substantial proportions of assisted patients are autonomous, have no comorbidities, are not polymedicated and have few comorbidities. This should allow the multivariable adjustments to be effective. Furthermore, the in-hospital mortality rate is not associated to professional home services even after adjusting for these characteristics. Similarly, the ED outcomes do not differ when adjustments are made. Thus, these are indications that multivariable adjustments should be considered as effective.

Our study revealed a significant proportion of dependent patients lacking professional support. However, we were not able to document more precisely the causes of this lack of professional services use, notably in relation with socio-economic characteristics. Nevertheless, studies exploring the use of home services by elderly individuals point to a strong correlation with income level [15, 25]. Financial barriers exist. The cost’s impact of services on the household disposable income depend on the global income and social context. In France, various financial support systems assume part of the induced cost but administrative processes are complex resulting in a high rate of elderly individuals forgoing these administrative aids [17].

Finally, to consider “having difficulties coping at home” as one of the diagnostics justifying hospital admission relied on the local investigator’s judgment. That situation refers to an inadequate balance between the patient’s functional resources and the constraints and complications that returning home might precipitate. Although it gets used in an empirical and informal fashion as a motive for hospitalization, no precise definition exists of this notion. In consequence, no guarantee can be given of its reproducibility between practitioners so that a classification bias may have intervened and may have produced an under- or overestimation of the parameter. Those subjective decisions, in spite of a potential classification bias, have impacted for good the way patients have been taken care of and hospitalized, which points to the importance of the link between the daily care for dependent patients at home and the pathway followed by elderly patients once visiting EDs.

## Conclusion

In summary, in this sample of French elderly patients visiting the ED, having professional services at home was not associated to lower in-hospital mortality. Assisted patients had a lower risk of being admitted for « having difficulties coping at home » but similar risk of being admitted after ED visit. A prospective study of health trajectories of elderly patients from ambulatory care to EDs, investigating formal and informal home support might furnish a measure of avoidable admission and potential incurred benefits.

## Supplementary information

**Additional file 1: Table S5.** Clinical details and resource utilization for elderly patients visiting EDs according to professional home services’ presence. **Table S6.** Detailed Odds-ratios (OR) of patients’ outcome after ED visit when comparing elderly patients assisted by professional home services to patients without any professional support.

## Data Availability

The datasets generated and analyzed during the current study are not publicly available due to legal restrictions. The national legislation in France protects personal data and materials. There are data restrictions concerning the EPIGER study since they contain sensitive and identifying patient personal information. Therefore, before any data transfer a legal authorization has to be obtained from CNIL, the French data privacy authority.

## Abbreviations

ED: Emergency Department
ADL: Activities of daily living
