# Supplementary material for: Emergency department outcome of elderly patients assisted by professional home services, the EPIGER study
Source: BMC Geriatr. 2020 Sep 21;20:355. doi: 10.1186/s12877-020-01742-1 (PMC7507819; doi:10.1186/s12877-020-01742-1)
Supplement: Supplementary file 1 — Additional file 1: Table S5. Clinical details and resource utilization for elderly patients visiting EDs according to professional home services’ presence. Table S6. Detailed Odds-ratios (OR) of patients’ outcome after ED visit when comparing elderly patients assisted by professional home services to patients without any professional support. [file 12877_2020_1742_MOESM1_ESM.docx]

**Supplemental Table 5**. Clinical details and resource utilization for elderly patients visiting EDs according to professional home services’ presence.

| **Characteristics** | **All patients**  **N=1168**  **N(%)** | **No services**  **N=800**  **N(%)** | **Services**  **N=368**  **N(%)** | **P-value** |
| --- | --- | --- | --- | --- |
| **Severity signs**:  GCS, median (Q1;Q3)  Resuscitation Bay  Organ failure  Serious acute illness on arrival | 15(15;15)  82(7)  54(5)  169(15) | 15(15;15)  56(7)  35(4)  110(14) | 15(15;15)  26(7)  19(5)  59(16) | 0.118  0.972  0.551  0.343 |
| **Performed exams in the ED:**  None  Biology tests  Imaging tests  Specialist advice  Social worker advice | 104(9)  946(81)  758(65)  297(25)  15(1) | 86(11)  624(78)  494(62)  213(27)  8(1) | 18(5)  322(88)  264(72)  84(23)  7(2) | 0.001  <0.001  0.001  0.166  0.203 |
| **Treatment administered in the ED:**  Painkillers  Suture  Psychotropic  Resuscitation  Surgery  Use of Physical Constraint | 375(32)  57(5)  29(3)  24(2)  34(3)  15(1) | 250(31)  39(5)  19(2)  17(2)  25(3)  11(1) | 125(34)  18(5)  10(3)  7(2)  9(2)  4(1) | 0.355  0.990  0.727  0.803  0.521  0.685 |
| **Main Cause for ED visit:**  Cardiology  Neurology  Trauma  Infection  Pneumology  Oncology  Psychiatry  Digestive | 153(13)  96(8)  278(24)  135(12)  107(9)  18(2)  44(4)  97(8) | 105(13)  68(9)  194(24)  90(11)  68(9)  12(2)  26(3)  66(8) | 48(13)  28(8)  84(23)  45(12)  39(11)  6(2)  18(5)  31(8) | 0.943  0.589  0.562  0.648  0.259  0.875  0.177  0.940 |

**Supplemental Table 6**. Detailed Odds-ratios (OR) of patients’ outcome after ED visit when comparing elderly patients assisted by professional home services to patients without any professional support.

| **Variable** | **Admitted** | **ED short unit** | **Intensive Care Unit** | **Coping difficulties** | **30 days admissions** |
| --- | --- | --- | --- | --- | --- |
| Professional home services | 0.92[0.65-1.30] | 0.81[0.57-1.14] | 0.70[0.30-1.64] | 0.59[0.38-0.92] | 1.10[0.75-1.61] |
| Age | 1.00[0.97-1.04] | 1.03[0.99-1.06] | 0.94[0.87-1.02] | 1.00[0.96-1.05] | 1.02[0.98-1.06] |
| Sex | 1.21[0.90-1.62] | 1.55[1.14-2.12] | 1.12[0.57-2.17] | 1.42[0.93-2.15] | 1.61[1.12-2.32] |
| ≥ 3 daily medications | 1.17[0.87-1.56] | 1.30[0.95-1.77] | 0.49[0.25-0.97] | 1.03[0.67-1.58] | 0.76[0.53-1.08] |
| Psychotropic drug | 1.36[0.94-1.96] | 1.18[0.83-1.67] | 1.17[0.52-2.66] | 1.50[0.97-2.33] | 0.96[0.64-1.43] |
| CV Disease history | 1.16[0.87-1.56] | 0.85[0.62-1.16] | 3.00[1.26-7.17] | 0.97[0.63-1.51] | 1.07[0.73-1.56] |
| Respiratory history | 1.28[0.86-1.90] | 1.56[1.08-2.27] | 0.45[0.16-1.25] | 0.95[0.56-1.59] | 0.58[0.36-0.94] |
| Neurological history | 1.08[0.74-1.58] | 1.23[0.85-1.77] | 1.87[0.85-4.12] | 0.84[0.52-1.35] | 1.06[0.69-1.60] |
| Cognitive Impairment | 0.97[0.66-1.42] | 1.13[0.78-1.63] | 0.34[0.12-0.96] | 1.80[1.15-2.82] | 1.00[0.66-1.51] |
| Knaus C | 3.26[1.99-5.34] | 1.38[0.85-2.24] | 0.41[0.12-1.37] | 10.5[4.91-22.58] | 1.16[0.66-2.03] |
| Imaging | 2.84[2.13-3.78] | 0.92[0.67-1.25] | 3.87[1.54-9.74] | 1.25[0.80-1.96] | 1.39[0.93-2.08] |
| Serious acute illness | 5.90[3.22-10.82] | 1.34[0.90-2.00] | 11.20[5.67-22.17] | 1.19[0.97-2.33] | 0.90[0.58-1.39] |
